# Resistant Starch Contents of Native and Heat-Moisture Treated Jackfruit Seed Starch

**DOI:** 10.1155/2015/519854

**Published:** 2015-01-06

**Authors:** Ornanong S. Kittipongpatana, Nisit Kittipongpatana

**Affiliations:** Department of Pharmaceutical Sciences, Faculty of Pharmacy, Chiang Mai University, Suthep Road, Mueang Chiang Mai District, Chiang Mai 50200, Thailand

## Abstract

Native jackfruit seed starch (JFS) contains 30% w/w type II resistant starch (RS2) and can potentially be developed as a new commercial source of RS for food and pharmaceutical application. Heat-moisture treatment (HMT) was explored as a mean to increase RS content of native JFS. The effect of the conditions was tested at varied moisture contents (MC), temperatures, and times. Moisture levels of 20–25%, together with temperatures 80–110°C, generally resulted in increases of RS amount. The highest amount of RS (52.2%) was achieved under treatment conditions of 25% MC and 80°C, for 16 h (JF-25-80-16). FT-IR peak ratio at 1047/1022 cm^−1^ suggested increases in ordered structure in several HMT-JFS samples with increased RS. SEM showed no significant change in the granule appearance, except at high moisture/temperature treatment. XRD revealed no significant change in peaks intensities, suggesting the crystallinity within the granule was mostly retained. DSC showed increases in *T*_*g*_ and, in most cases, Δ*T*, as the MC was increased in the samples. Slight but significant decreases in Δ*H* were observed in samples with low RS, indicating that a combination of high moisture and temperature might cause partial gelatinization. HMT-JFS with higher RS exhibited less swelling, while the solubility remained mostly unchanged.

## 1. Introduction

Seeds of jackfruit (*Artocarpus heterophyllus* Lam.), considered as biowaste by the canned food industry, are recognized by many research studies as a candidate for a new source of commercial starch [1–4]. Physicochemical, functional, and pharmaceutical properties of jackfruit seed starch (JFS) and its physically and chemically modified starches have been widely studied and the results suggested potential applications in food, functional food, and pharmaceutical products [5–9]. However, the utilization of JFS in such products remained very limited, partly due to a higher production cost but mainly because similar properties or functionality could be obtained from existing commercial starches, that is, corn, cassava, and potato. The attempt to increase the use of JFS thus required more studies in the aspects that have not previously been explored, for example, enzyme modification and resistant starch content of JFS.

Jackfruit seeds have long been used as foods among local people in many areas of the world [2]. The nutrition values of the seeds, which contained an average of 20–25% starch, have been well documented. Because of a relatively high amylose content (24–32%), native JFS could contain a reasonable amount of type II resistant starch (RS2). RS, defined as the sum of starch and starch products not hydrolyzable in the small intestine, has gained considerable attention in recent years due to its reported benefits to the GI system in similar fashion to the dietary fiber [10]. RS is now a common additive in foods and functional foods [11], and many research studies suggest the expanded utilization into the pharmaceutical industry where RS can serve as a part of a drug delivery system to the colon [12, 13] in addition to the health benefit as active ingredient.

A number of naturally high-RS, native starches are reported in the literature [11], although most of these starches are from rare sources which prevented them from being commercialized in the near future. Commercial RS2 currently available is mainly high-amylose corn starch and potato starch. There are also a number of studies that report the production and increase of RS from commercial starch sources such as rice, cassava, and mung bean starches by several techniques, including enzyme debranching and chemical and hydrothermal modification [14, 15]. Heat-moisture treatment (HMT) is a hydrothermal modification method that has commonly been explored to alter the physicochemical, digestibility, and functional properties of starch with minimum effect on the granule structure [16]. Typical HMTs are carried out at moisture content of 35% w/w or below and at temperatures between the glass transition and the gelatinization temperatures, with the exposure time up to 16 h [15, 17]. HMT brings about structural stability due to the rearrangement of amylose chains into more-ordered domain, which also results in changes in granular swelling, gelatinization temperature, and, in many cases, RS content. These effects, however, were reported to vary from one starch source to another, as starches from different botanical sources exhibited different responses to HMT conditions [18].

The objectives of this study were to determine the RS content in JFS compared to other common native starches and a commercial RS sample and to investigate the effects of HMT on the properties and RS contents of JFS. Results presented in this paper will facilitate the study of JFS as a new source of resistant starch for food and possible pharmaceutical industry.

## 2. Experimental Section

### 2.1. Materials

Seeds of jackfruit cultivar “Thong Prasert” were obtained as a single lot (20 kg). Preparation of jackfruit seed flour was carried out using lye-peel method as described by Tulyathan et al. [4]. JFS was then extracted from the flour using a method described previously [5]. Hi-maize 260 (National Starch Food Innovation, USA) was a gift from Food & Cosmetic Systems Co. Ltd. (Bangkok, Thailand). Mung bean starch was obtained from Sitthinan Co. Ltd. (Bangkok, Thailand). Rice starch was purchased from Thai Flour Industry Co. Ltd. (Bangkok, Thailand). Potato starch was supplied by Continental Food Co. Ltd. (Bangkok, Thailand). Banana starch was extracted from 4-week-old raw banana fruits using a method described by Waliszewski et al. [19].

### 2.2. Heat-Moisture Treatment of JFS

The moisture content (MC) of JFS, initially determined to be 10.3%, was adjusted by adding water to obtain starch samples with moisture contents of 20, 25, 30, and 35% w/w, respectively. A 25 g portion of each sample, along with the native JFS, was placed in a hermetically sealed stainless steel container and heated in a hot-air oven set at 80, 90, 100, 110, and 120°C for 6, 12, and 16 hours. A total of 75 HMT-JFS samples were dried in a hot-air oven at 40°C for 48 h into uniform moisture content (~10–12%) and ground in a mortar to pass through an 80-mesh screen. The obtained products were assigned codes as JF-[% MC]-[temperature (°C)]-[time (h)].

### 2.3. Determination of Resistant Starch Content

Resistant starch (RS) content in samples was determined using a Megazyme Resistant Starch Assay Kit (AOAC Method 2002.02). In brief, a screw-capped test tube containing 100 mg sample and 4.0 mL solution of pancreatic *α*-amylase (10 mg/mL, pH 6.0) and amyloglucosidase (3 U/mL) was incubated in a shaking water bath at 37°C for 16 h. The reaction was stopped with 4 mL ethanol and centrifuged at 4000 g for 10 min to separate the digested (supernatant) part from the nondigested (residue) part. The supernatant was diluted with 100 mM sodium acetate buffer. An aliquot of the solution was incubated with amyloglucosidase (10 *μ*L, 300 U/mL) at 50°C for 20 min. The residue was dissolved in 2 M KOH (2 mL) in an ice bath, added with 1.2 M sodium acetate buffer (8 mL), and hydrolyzed to glucose with amyloglucosidase (0.1 mL, 3300 U/mL) at 50°C for 30 min. The glucose oxidase/peroxidase (GOPOD) reagent was added to the aliquot portion of each part, incubated at 50°C for 20 min. Absorbance was then measured at 510 nm. Resistant starch and nonresistant (digested) starch were calculated as glucose × 0.9. The total starch was calculated as the sum of resistant and digested starch. Because values of RS content were reported to vary among different methods of determination [20], RS contents of banana starch, cassava starch, mung bean starch, rice starch, and a commercial RS starch, Hi-maize 260, were also determined under the same condition for comparison purpose.

### 2.4. Amylose Content

Amylose contents (AC) of JFS, HMT-JFS, and other starches were determined using a colorimetric method based on a complexation between starch and iodine according to Juliano [21].

### 2.5. Scanning Electron Microscopic (SEM) Analysis

SEM experiments to analyze the granule surface, shape, and size were conducted using a JEOL instrument model JSM-5410LV (JEOL, USA) equipped with a large field detector. The acceleration voltage was 15 kV under low vacuum mode (0.7-0.8 torr). The sample was placed on a copper stub covered with adhesive tape and coated with gold under vacuum. The images were taken at 2000x magnification.

### 2.6. X-Ray Diffraction (XRD)

XRD patterns were recorded in the reflection mode on a Siemens D-500 X-ray diffractometer. Diffractograms were registered at Bragg angle (2*θ*) range of 5–40° at a scan rate of 2.5°/min and step size of 0.02°.

### 2.7. Thermal Properties

Thermal properties were assessed using a Perkin Elmer DSC-7 differential scanning calorimeter. The analysis was carried out at a temperature between 30 and 120°C, at 10°C/min, on a 1 : 3 (w/w) starch-water mixture sample. An empty pan was used as a reference. The temperatures of the characteristic transitions, onset (*T*_*o*_), peak (*T*_*p*_), and conclusion (*T*_*c*_) temperatures, were recorded and the gelatinization temperature ranges (*T*_*c*_–*T*_*o*_, Δ*T*) were calculated. Enthalpy change of gelatinization (Δ*H*) was calculated and expressed as J/g of dry starch.

### 2.8. Attenuated Total Reflectance Fourier-Transformed Infrared Spectroscopy (ATR-FT-IR)

FT-IR spectra were recorded on a Nicolet Nexus 470 FT-IR equipped with a DTGS detector using an attenuated total reflectance (ATR) mode. For each spectrum, 64 scans were recorded at a resolution of 4 cm^−1^. Spectra were baseline-corrected using Omnic version 6.2. The region at 1200–800 cm^−1^ was deconvoluted and the absorbance values at 1047 and 1022 cm^−1^ were determined using PeakFit version 4.12 software. The peak ratio of 1047/1022 cm^−1^, a parameter used to quantitatively characterize the degree of order and structural changes, was calculated for each sample.

### 2.9. Swelling Power and Water Solubility

Sample (0.1 g) was placed into each of five preweighed centrifuge tubes containing 10 mL water, mixed thoroughly for 1 min, and then heated at controlled temperatures of 50, 60, 70, 80, and 90°C, respectively, with regular stirring. After 10 min, the tubes were cooled and centrifuged at 3000 rpm for 15 min. The supernatant was dried to a constant weight at 120°C. The weights of the dried residue and of the sedimented paste were used to calculate the solubility percentage and the swelling power, respectively [5].

### 2.10. Statistical Analysis

All tests were performed at least in triplicate. The statistical significant tests were performed using analysis of variance (ANOVA) at 95% confidence level (*P* < 0.05). Significant differences among mean values were determined by Duncan's multiple range test.

## 3. Results and Discussion

### 3.1. Resistant Starch Contents and Amylose Contents

#### 3.1.1. Native JFS versus Other Starches and Commercial RS

Under the same analytical conditions, the RS2 content in native JFS was much higher than that of mung bean starch, cassava starch, and rice starch, but it remained significantly lower than that of raw banana starch and Hi-maize 260 starch (Table 1). Hi-maize, a high-amylose corn starch, and banana starch are known for their high RS content [11, 22]. The value for mung bean starch was slightly higher than that reported by [15] (11.2 ± 0.1%), using essentially the same conditions. This could partly be due to the higher amylose content (35%) of mung bean starch used in this study, as compared to that of material used in the previous report (29.7%). Differences in RS content among starches from various botanical sources were due not only to the chemical/compositional parameters (e.g., amylose and PO_4_ contents) but also to the physical/structural (i.e., granule shape and size, crystallinity pattern, molecular interaction, and arrangement) characteristics of each starch [11].

#### 3.1.2. HMT Jackfruit Seed Starches

**Figure 1:**
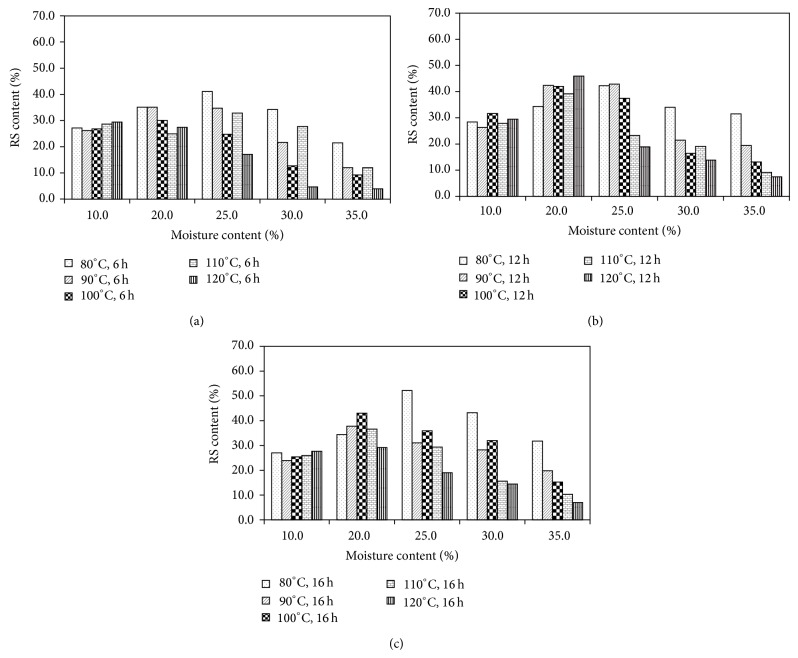
Resistant starch contents of heat-moisture treated (HMT) jackfruit seed starch samples. Treatment conditions were 10–35% MC and 80–120°C, for (a) 6 h, (b) 12 h, and (c) 16 h.

Upon HMT, the RS contents varied considerably. No significant change in RS content, compared to non-HMT sample, was observed for the 10% MC samples after incubation at various temperatures and times, indicating that there was a minimum amount of moisture required for starch granules to undergo transition. In HMT-JFS samples prepared at 20% MC, RS content increased in samples treated at 80–100°C for 6 h, at 80–120°C for 12 h, and at 80–110°C for 16 h. Samples adjusted to 25% MC before HMT showed significant increases in RS content when the conditions were 80–90°C for 6 h or 80–100°C for 12 or 16 h. Most of these samples exhibited RS contents in the range of 35 to 45%. The highest RS content, 52.2%, was achieved in a sample adjusted to 25% MC and incubated at 80°C for 16 h (JF-25-80-16). In this case, however, high RS contents observed in JF-25-80-6 and JF-25-80-12 suggested that the time of treatment might have lesser effect than MC and temperature. At 30% MC, samples treated at 80°C had slight increases in RS content, while higher temperatures started to show some diminished RS content. At 35% MC, decreases in RS content were observed, especially at higher temperatures and longer periods of heat exposure. The sample JF-35-120-16 showed the lowest RS content at 7.0% (Figure 1). Similar results were reported in faba bean, in which the RS content was increased upon HMT at 80°C but then significantly decreased when the temperature was 120°C [23]. Both the moisture content and the temperature used in HMT could affect the organization of the crystalline portions in the starch granules by allowing more access of the enzymes into the granules. The decrease in RS content observed in samples treated at 30–35% MC and high (100–120°C) temperatures could also be a result of partial gelatinization [24].

**Figure 2:**
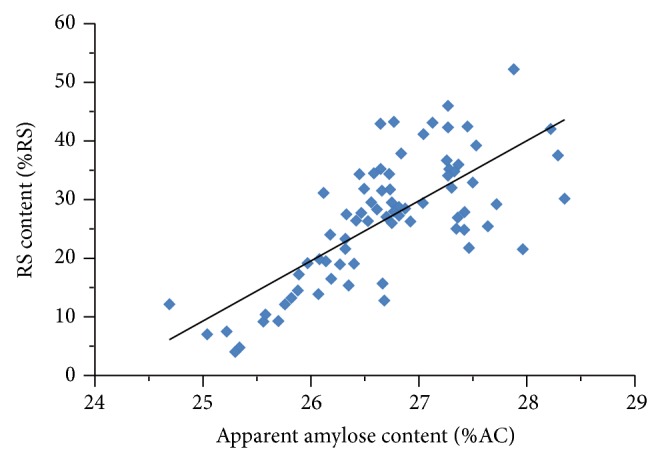
Relationship between amylose content and resistant starch contents in HMT-JFS.

Apparent amylose content of HMT-JFS samples ranged between 24.7 and 28.4%. AC average of 26.7 ± 0.8%, on first glance, was not significantly different from that of native starch. A closer look at the relationship between AC and RS content, however, revealed that there possibly was a correlation between the two parameters in HMT samples. Samples with higher AC were likely to have higher RS content. Linear regression analysis of a plot between AC and RS content (Figure 2) yielded a correlation coefficient (*r*) of 0.73. Increases in AC in HMT samples resulting in higher RS were also reported for mung bean starch [15] and were suggested to be a result of the interaction between starch chains within the amorphous area of the granule. RS content was also reported to be higher in starch with higher AC, when starch from the same botanical species was used [25]. The decrease in AC upon HMT was proposed to be due to heat-induced change on amylose conformation which restricted the ability of amylose to form longer or more-ordered helical segments, thus decreasing the ability of amylose to form a complex with iodine.

### 3.2. SEM Analysis

**Figure 3:**
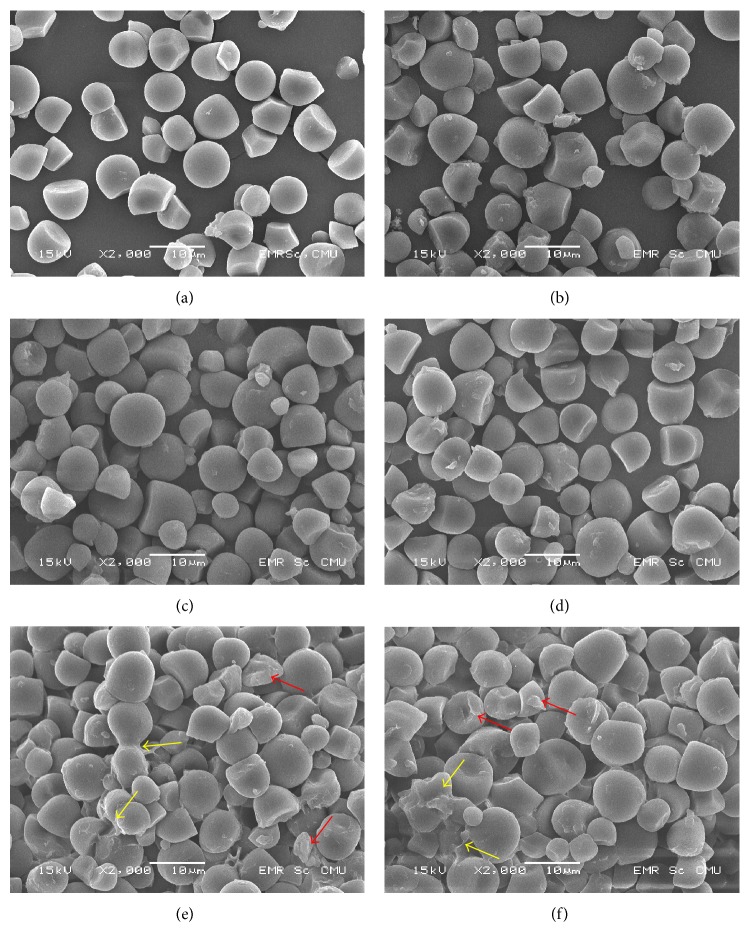
Scanning electron microscopic (SEM) images of JFS and representative HMT-JFS. (a) Native JFS, (b) JF-N-80-6, (c) JF-20-90-12, (d) JF-25-100-16, (e) JF-30-110-12, and (f) JF-35-120-16.

SEM images of native JFS and some HMT-JFS are presented in Figure 3. HMT-JFS samples subjected to low/medium moisture contents (10–25%) and temperatures (80–100°C) treatment showed no significant change in the granule morphology compared to that of native JFS (Figures 3(a)–3(d)). At higher moisture (30–35%) and temperature (110–120°C) treatment (Figures 3(e) and 3(f)), granules appeared to be more swollen with some of the round and bell-like granules becoming more irregular (red arrows). Granule fusion and surface corrosion were also observed (yellow arrows). This is likely caused by partial gelatinization brought about by a combination of high moisture and heat in HMT [26, 27].

### 3.3. XRD

**Figure 4:**
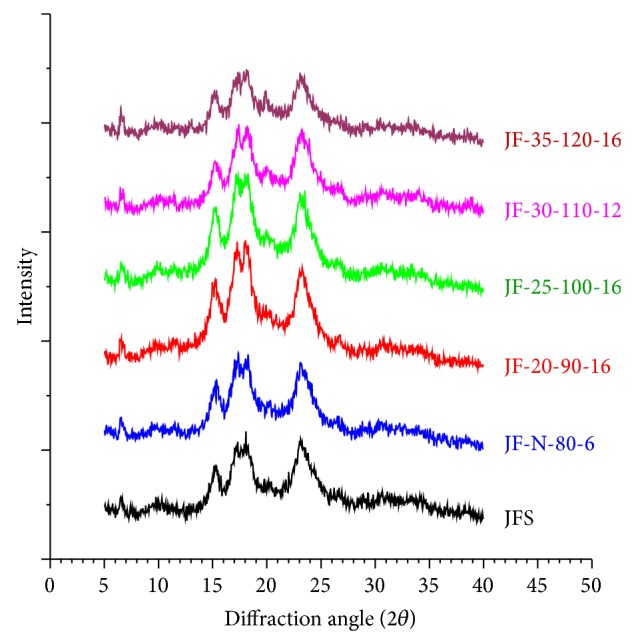
X-ray diffractograms of native JFS and five representative HMT-JFSs.

XRD pattern of JFS showed strong diffraction peaks at Bragg angles 2*θ* of 15.3°, 17.2°, 18.1°, and 23.1°, consistent with an A-type crystallinity pattern, as reported previously [4, 5]. After HMT, all samples retained A-type crystalline pattern, but with different peak intensities. The treatment with 10% MC at all temperatures and times yielded samples which showed virtually identical XRD pattern and peak intensities to those of JFS. Samples treated with medium MC and temperatures showed increased intensity of all reflection peaks, suggesting a more-ordered rearrangement within the granules. In contrast, samples treated with high MC and temperature showed slightly (for 30% MC) or significantly (for 35% MC) decreased intensity of reflection peaks (Figure 4), congruent with the changes on the granule surface observed in SEM results, and further confirmed the explanation that high moisture and temperature facilitated destabilization of lamellar array [18].

### 3.4. Thermal Properties

**Figure 5:**
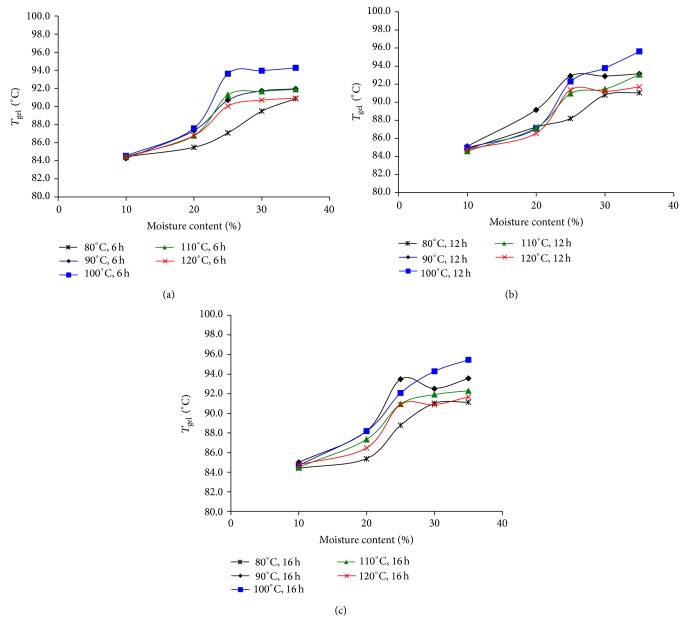
Gelatinization temperatures of HMT-JFS samples prepared under varied conditions of moisture contents (10–35%), temperatures (80–120°C), and times (6, 12, and 16 h).

A relatively high gelatinization temperature (*T*_*g*_) of native JFS (84.24 ± 0.37°C) suggested, in part, that the structural organization within the granules was more ordered as compared to other starches with lower *T*_*g*_. Increases in gelatinization parameters (*T*_*o*_, *T*_*g*_, and *T*_*c*_) were observed in 16 h HMT-JFS samples and are presented in Table 2. This was in agreement with other HMT starches [18, 27, 28] which suggested reduced mobility of starch chain within amorphous region caused by structural changes within starch granule due to amylose-amylose, amylose-amylopectin, and/or amylose-lipid interactions [28]. Increases also occurred similarly in 6 and 12 h HMT-JFS samples. Samples treated at higher % MC showed higher *T*_*g*_ (Figure 5), although no correlation with the change in RS content in the samples was observed. Slight to moderate decreases in Δ*T* (*T*_*g*_ range) were seen in some HMT-JFS samples treated at 80–100°C. Similar results have been reported for rice, cassava, and pinhão starches [18]. Samples treated at higher temperatures showed increased Δ*T*, which was typical for HMT [14, 16]. Decreases in the gelatinization enthalpy (Δ*H*) were slight to moderate (0.5–4.0 J/g) in samples treated with 20–25% MC at 80–100°C. At higher MC and/or temperatures, the decrease in Δ*H* values was more pronounced (5.5–9.5 J/g). Decreased or unchanged Δ*H* of starches upon HMT was common [14, 16, 18, 23, 26] as a result of the disruption of hydrogen bonds among double helices in the crystalline and noncrystalline regions of starch granule due to heat-induced, increased mobility. It could also be due to partial gelatinization caused by a combination of high moisture and temperature [27]. However, increased Δ*H* values after HMT have been reported in recent studies on mung bean starch [15] and rice starch and flour [26]. Such increases were proposed to be due to greater amounts of double helices or stronger interaction between starch chains within the crystalline domains [15].

### 3.5. Attenuated Total Reflectance Fourier-Transformed Infrared Spectroscopy (ATR-FT-IR)

The ratio of absorbance 1047/1022 cm^−1^ of several HMT-JFS samples was similar to or higher than that of native starch (0.81–0.85 versus 0.80) (Table 2), indicating that the external regions of granules of these HMT-JFSs were more organized as a result of increases in ordered structure and could explain the increased RS content in these samples [15]. On the other hand, decreases in ordered structure in high MC and high temperature-treated samples were reflected by the lowering of 1047/1022 cm^−1^ peak ratio (0.70–0.78) in these samples. The values, however, were not as low as that of pregelatinized starch (0.63–0.65), in which most of the granules were ruptured and the crystallinity was destroyed, but were more similar to that of carboxymethyl starches prepared using 2-propanol as solvent (i-CMJF) (0.74–0.76), in which partial gelatinization was evident but the granules retained their integrity and crystallinity [5]. The peak ratio at 1047/1022 cm^−1^ has been used as a parameter to assess structural organization or change of starch chains on a molecular level [28] and was shown to correlate with RS content in HMT starches of faba bean, black bean, and pinto bean [23].

### 3.6. Swelling Power and Water Solubility

**Figure 6:**
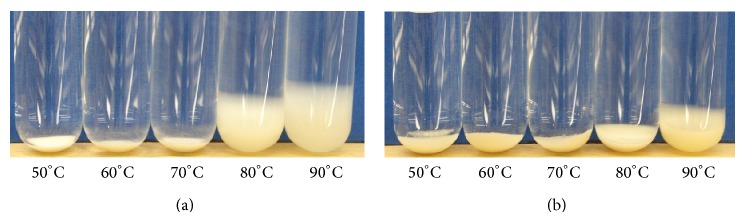
Swellability of (a) native JFS and (b) a representative HMT-JFS (JF-25-80-16), at various (50–90°C) temperatures.

Swelling of native and HMT-JFS was temperature dependent. JFS showed very slight swelling in water up to 70°C. At 80°C, JFS showed significant increase in swelling and continued to increase as the temperature was raised to 90°C. Madruga et al. [2] reported similar swelling profiles for soft and hard jackfruit seed starch, with significant swelling starting at above 75°C and reaching maximum values of 15–18 g/g at 85–95°C. HMT-JFS exhibited less swelling at 50–70°C and showed only slight and moderate swelling at 80 and 90°C, respectively (Figure 6). Different HMT conditions yielded different swelling power, although no correlation between the two parameters was observed. The decrease in swelling power was suggested to be a result of the rearrangement within starch granule structures, the reduction of hydration, and/or induced amylose-amylose and amylose-amylopectin interactions upon HMT [18, 29]. The results were also consistent with previous reports on mung bean [15], corn [28], faba bean, clack bean, and pinto bean [23] starches.

The effect of HMT on water solubility, on the other hand, was not obvious as HMT-JFS samples exhibited mostly similar or slightly decreased water solubility compared to that of JFS. An only exception was for HMT samples at 100°C in which the decrease in solubility was significant in samples of higher MC (Table 2). Previous studies reported reduced solubility in some HMT starches, including African yam bean [30], rice, cassava and pinhão [18], rice [29], and sorghum [31] starches, while increased solubility was found in HMT starches of mung bean [15] and finger millet [32]. It was reported that changes in physical properties of HMT starches including granular appearance, XRD pattern, swelling power, and solubility, as well as thermal properties, varied extensively due to the sources of starch and HMT conditions [15].

One major concern regarding the practical significance of resistant starch type II in the food industry is that the RS content of raw starch is greatly reduced or almost eliminated when the starch is cooked. However, a number of studies have recently reported applications of RS in food products, with some potential values. Examples include ice cream added with RS, which acts as a prebiotic compound [33], and yogurts containing resistant starch [34]. Applications in the pharmaceutical industry are more practical as pharmaceutical excipients or adjuvants, since many processes in pharmaceutical dosage form manufacturing do not involve high heat. RS has been reported as potential excipients in the colon drug delivery system [12].

## 4. Conclusions

Jackfruit seed offers a sustainable source of potentially new commercial starch, with comparatively high amount of resistant starch. Heat-moisture treatment was shown to be an effective mean to increase resistant starch content in jackfruit seed starch. The moisture content and the temperature of treatment significantly affected the resistant starch content, while the time of treatment seemed to have lesser effect. The moisture levels of 20–25%, together with temperature between 80 and 110°C, generally resulted in increases of RS amount, while higher moisture contents and/or temperatures led to drastic decreases. Under an optimum condition, the obtained RS amount was comparable to that of commercial resistant starch. Moderate changes in SEM and XRD profiles were observed in samples treated with high moisture/temperature. DSC analyses showed increases in the gelatinization temperature as the MC was increased in the samples. Amylose contents changed in a narrow range but exhibited a correlated trend with RS content. Samples with higher RS exhibited less swelling, while the solubility remained mostly unchanged. This starch source and HMT technique could collectively be used to prepare commercial resistant starch, currently in demand for food and pharmaceutical industries.

## Figures and Tables

**Table 1:** Resistant starch and amylose contents of JFS and other native starches compared to a commercial RS.

Starch	RS (%)	AC (%)
Jackfruit seed	29.7 ± 2.4	26.4 ± 0.7
Banana starch	58.7 ± 2.1	27.9 ± 0.4
Cassava starch	7.3 ± 1.1	23.3 ± 0.6
Mung bean starch	14.5 ± 0.9	35.1 ± 0.6
Rice starch	1.1 ± 0.3	21.2 ± 0.8
Hi-maize 260	41.3 ± 0.7	52.0 ± 1.3

**Table 2:** Thermal properties, FT-IR ratio of peaks 1047/1022 cm^−1^, swelling power (g/g of dry starch), and water solubility (g/g of dry starch × 100) of native JFS and selective HMT-JFS treated at conditions of 10–35% MC and 80–120°C for 16 h.

JFS sample	Temperature (°C)			Δ*T*	Δ*H*	FT-IR ratio	Swelling power at *T* (°C)						Solubility at *T* (°C)					
	*T*_*o*_	*T*_*p*_	*T*_*c*_		(J/g)		RT	50	60	70	80	90	RT	50	60	70	80	90
Native	82.58 ± 0.24	84.24 ± 0.37	88.63 ± 0.12	6.05 ± 0.31	11.25 ± 0.06	0.80 ± 0.01	1.1	1.3	2.3	2.5	6.8	7.7	0.4	1.3	2.4	2.8	7.2	9.5

80°C																		
10% MC	82.28 ± 0.94	84.41 ± 0.54	88.75 ± 0.07	6.47 ± 0.37	10.14 ± 0.06	0.82 ± 0.03	1.1	1.3	2.1	2.9	5.3	7.0	0.5	1.5	2.2	2.9	7.2	9.3
20% MC	82.52 ± 0.31	85.38 ± 0.24	88.25 ± 0.21	5.73 ± 0.10	7.44 ± 0.26	0.85 ± 0.02	1.0	1.4	2.3	2.9	5.0	6.7	0.7	1.5	2.3	3.1	7.1	9.2
25% MC	86.10 ± 0.12	88.79 ± 0.39	91.65 ± 1.06	5.55 ± 0.94	7.27 ± 0.73	0.84 ± 0.02	1.1	1.5	2.5	2.8	3.2	5.7	0.7	1.7	2.3	3.2	7.3	9.1
30% MC	89.22 ± 0.27	91.03 ± 0.13	93.25 ± 0.21	4.03 ± 0.06	4.49 ± 0.23	0.83 ± 0.03	1.1	1.2	2.4	2.7	4.3	5.5	0.6	1.6	2.5	3.0	7.2	9.2
35% MC	89.32 ± 0.14	91.14 ± 0.16	93.40 ± 0.71	4.08 ± 0.85	5.04 ± 0.36	0.85 ± 0.04	1.2	1.2	2.6	2.6	5.0	6.1	0.5	1.7	2.6	3.1	7.3	9.6

90°C																		
10% MC	82.14 ± 1.20	85.01 ± 0.63	88.05 ± 0.92	5.91 ± 0.28	9.20 ± 0.95	0.78 ± 0.06	1.0	1.2	2.3	3.1	5.9	7.3	0.4	1.4	2.3	2.9	6.8	9.1
20% MC	85.71 ± 0.09	88.20 ± 0.40	91.90 ± 1.41	6.19 ± 1.51	8.12 ± 1.10	0.84 ± 0.04	1.1	1.5	2.7	3.3	5.5	6.9	0.4	1.5	2.4	3.2	7.0	9.3
25% MC	91.62 ± 0.20	93.49 ± 0.55	96.20 ± 1.13	4.58 ± 0.93	7.41 ± 1.12	0.77 ± 0.05	1.1	1.4	2.6	2.9	5.6	6.7	0.3	1.6	2.4	3.1	6.8	9.2
30% MC	90.57 ± 0.23	92.53 ± 0.16	95.00 ± 0.57	4.43 ± 0.80	5.11 ± 0.99	0.78 ± 0.06	1.0	1.3	2.5	3.2	5.3	6.2	0.4	1.5	2.6	3.1	7.0	9.3
35% MC	91.35 ± 0.48	93.57 ± 0.56	96.50 ± 1.56	5.15 ± 1.08	4.87 ± 1.37	0.75 ± 0.05	1.0	1.4	2.7	2.9	5.4	6.6	0.4	1.8	2.6	3.0	7.0	9.5

100°C																		
10% MC	81.68 ± 0.44	84.69 ± 0.45	89.00 ± 0.85	7.32 ± 0.41	10.75 ± 0.33	0.78 ± 0.03	1.1	1.3	2.2	3.1	5.6	6.5	0.5	1.2	2.3	3.0	6.6	8.9
20% MC	85.37 ± 0.32	88.18 ± 0.51	91.75 ± 1.20	6.38 ± 0.88	8.93 ± 1.04	0.76 ± 0.03	1.1	1.2	2.3	3.2	5.2	5.8	0.4	1.1	2.3	2.9	5.7	8.2
25% MC	89.13 ± 0.27	92.07 ± 0.03	95.25 ± 0.21	6.12 ± 0.06	5.80 ± 0.93	0.76 ± 0.05	1.0	1.2	2.5	2.9	4.8	5.4	0.5	1.1	2.4	2.8	5.0	7.5
30% MC	92.12 ± 0.09	94.29 ± 0.05	97.60 ± 0.00	5.48 ± 0.09	4.38 ± 0.64	0.75 ± 0.03	1.0	1.1	2.2	2.8	4.0	5.3	0.5	1.3	2.3	2.7	4.7	7.2
35% MC	93.26 ± 0.01	95.45 ± 0.07	98.05 ± 0.04	4.79 ± 0.22	1.80 ± 0.06	0.70 ± 0.05	1.1	1.1	2.2	2.5	3.3	4.6	0.4	1.1	2.2	2.6	4.4	6.9

110°C																		
10% MC	81.66 ± 0.38	84.50 ± 0.27	88.25 ± 0.21	6.59 ± 0.59	9.35 ± 1.33	0.81 ± 0.01	1.0	1.3	2.3	3.2	5.8	7.2	0.3	1.3	2.4	3.4	7.0	9.0
20% MC	84.92 ± 0.90	87.34 ± 0.15	91.55 ± 0.21	6.63 ± 1.12	3.10 ± 1.36	0.84 ± 0.02	1.0	1.3	2.4	3.2	5.4	6.8	0.3	1.3	2.5	3.5	7.2	8.8
25% MC	86.49 ± 1.25	90.95 ± 0.12	94.80 ± 0.85	8.31 ± 0.40	4.55 ± 0.84	0.77 ± 0.05	1.2	1.6	2.7	3.3	5.5	6.5	0.4	1.2	2.5	3.4	7.2	8.7
30% MC	89.21 ± 0.67	91.92 ± 0.66	95.75 ± 0.92	6.54 ± 0.25	4.75 ± 1.20	0.76 ± 0.03	1.2	1.4	2.7	3.4	4.9	6.1	0.4	1.3	2.6	3.7	7.3	8.9
35% MC	89.29 ± 0.17	92.31 ± 0.18	95.55 ± 0.21	6.26 ± 0.05	1.63 ± 0.07	0.78 ± 0.04	1.2	1.5	2.8	3.6	4.8	6.5	0.4	1.4	2.5	3.6	7.6	9.4

120°C																		
10% MC	81.75 ± 0.72	84.76 ± 0.73	89.05 ± 2.19	7.30 ± 1.47	9.29 ± 0.93	0.82 ± 0.02	1.1	1.2	2.2	3.2	5.7	7.0	0.5	1.6	2.2	3.3	7.4	9.2
20% MC	86.05 ± 4.47	86.48 ± 0.21	90.65 ± 0.07	6.60 ± 0.54	4.14 ± 0.25	0.82 ± 0.03	1.1	1.3	2.4	3.3	5.9	6.8	0.4	1.6	2.3	3.4	7.3	9.3
25% MC	87.33 ± 2.10	90.91 ± 0.14	93.90 ± 0.57	6.57 ± 0.66	3.88 ± 0.90	0.77 ± 0.05	1.3	1.4	2.5	2.9	5.5	6.6	0.5	1.8	2.3	3.6	7.5	9.6
30% MC	86.92 ± 0.27	90.88 ± 0.96	95.80 ± 0.99	8.88 ± 1.26	3.18 ± 0.74	0.74 ± 0.04	1.3	1.5	2.6	3.1	5.5	6.9	0.5	1.9	2.5	3.5	7.4	9.4
35% MC	88.27 ± 0.16	91.68 ± 0.19	94.75 ± 0.35	6.48 ± 0.19	2.28 ± 0.35	0.69 ± 0.03	1.2	1.5	2.6	3.4	5.8	7.2	0.5	1.6	2.6	3.3	7.5	9.5

## Abbreviations

JFS: Jackfruit seed starch
HMT: Heat-moisture treatment
RS: Resistant starch.
